# Structural Comparison of hMDH2 Complexed with Natural Substrates and Cofactors: The Importance of Phosphate Binding for Active Conformation and Catalysis

**DOI:** 10.3390/biom12091175

**Published:** 2022-08-25

**Authors:** Yumi Eo, Men Thi Hoai Duong, Hee-Chul Ahn

**Affiliations:** College of Pharmacy, Dongguk University-Seoul, Goyang, Gyeonggi 10326, Korea

**Keywords:** malate dehydrogenase 2, MDH2, crystal structure, TCA cycle, malate, oxaloacetate, NAD, NADH, phosphate, isothermal titration calorimetry

## Abstract

Malate dehydrogenase (MDH), which catalyzes a reversible conversion of _L_-malate to oxaloacetate, plays essential roles in common metabolic processes, such as the tricarboxylic acid cycle, the oxaloacetate–malate shuttle, and the glyoxylate cycle. MDH2 has lately been recognized as a promising anticancer target; however, the structural information for the human homologue with natural ligands is very limited. In this study, various complex structures of hMDH2, with its substrates and/or cofactors, were solved by X-ray crystallography, which could offer knowledge about the molecular and enzymatic mechanism of this enzyme and be utilized to design novel inhibitors. The structural comparison suggests that phosphate binds to the substrate binding site and brings the conformational change of the active loop to a closed state, which can secure the substate and cofactor to facilitate enzymatic activity.

## 1. Introduction

Malate dehydrogenases (EC 1.1.1.37) catalyze a reversible conversion between _L_-malate and oxaloacetate coupled with an oxidation/reduction of the co-enzymes NAD and NADH [1]. In eukaryotic cells, three types of NAD-dependent MDHs were found, including cytoplasmic, mitochondrial, and glyoxysomal MDHs. Mitochondrial MDH is included in the tricarboxylic acid (TCA) cycle [2] and cytoplasmic MDH, and mitochondrial MDH play roles in the malate–aspartate shuttle [3]. Glyoxysomal MDH is found in the glyoxysomes, which converts glyoxylate into malate in the glyoxylate cycle [4]. Some bacteria (e.g., *Escherichia coli*) possess the NAD-dependent MDH [5], which is similar in amino acid sequence to mitochondrial MDH, while other bacterial MDHs (e.g., *Thermus flavus*) are more similar to the cytoplasmic MDH [6]. The structural and biochemical characterizations of MDHs from diverse species have been extensively studied, since MDHs are responsible for numerous significant metabolic pathways [7].

The MDH is analogous to lactate dehydrogenase (LDH) in many aspects, such as tertiary structure, cofactor binding, and catalytic residues, although their substrate specificity is a distinct difference [8]. MDH selectively binds to di-carboxylic acid, malate, or oxaloacetate, whereas LDH binds to mono-carboxylic acid, lactate. Two positively charged arginine residues in MDH interact with di-carboxylic acid substrates [9], while glutamine in LDH avoids this ionic interaction [10].

The human mitochondrial malate dehydrogenase (hMDH2) has a molecular weight of 36 kDa and contains the N-terminal transit peptide (residues 1 to 24), enabling the protein to translocate to the mitochondria. The enzyme activity is enhanced by acetylation on some lysine residues (Lys185, Lys301, Lys307, and Lys314) [11]. It has been reported that citrate is an allosteric effector of mitochondrial MDH. Citrate inhibits the reduction in oxaloacetate under all conditions. Citrate also inhibits malate oxidation, but only at low malate or NAD concentrations; however, it enhances MDH activity in the presence of high levels of malate and NAD concentrations [12]. The crystal structure of *E. coli* MDH and the citrate complex where citrate is bound in the substrate binding site supported the allosteric effect of citrate on the enzymatic activity of MDH [13].

It has been considered that MDH2 is a potential molecular target for cancer therapy, and several chemical compounds have been reported as inhibitors of MDH2. Differentiation-inducing factor-1 (DIF-1) secreted from *Dictyostelium discoideum*, which was known to inhibit the proliferation of several cancer cells by downregulation of the Wnt/β-catenin signaling pathway, revealed its binding to MDH2 and inhibiting its activity. This implied that the inhibition of MDH2 activity by DIF-1 could be one of the mechanisms of reducing proliferation [14]. In a recent study, hMDH2 was identified as a target molecule of LW6, a hypoxia inducible factor (HIF)-1α inhibitor, suggesting that LW6 suppresses HIF-1α accumulation via inhibition of MDH2 activity, subsequently suppressing mitochondrial respiration. Consequently, the inhibition of MDH2 by LW6 results in decreased tumor growth [15].

Even though various MDH structures of different organisms were previously determined, the structures of hMDH2 or its complexes with the natural ligands or chemicals have thus far rarely been studied. In this study, we present six complex structures of hMDH2, with its substrates and/or cofactors solved by X-ray crystallography. The detailed comparison between the structures complexed with the substrate, with the substrate and the cofactor, and with the allosteric inhibitor is provided. This includes the specificity of hMDH2 to _L_-malate against _D_-malate and the slight structural differences and binding thermodynamics in cofactor binding. Finally, we propose that anions, such as phosphate, substrates, and substrate-mimics bind to the substrate binding site followed by the closing of the active loop, which can stabilize the substrate and cofactor to facilitate enzymatic activity.

## 2. Materials and Methods

### 2.1. Cloning, Protein Expression, and Purification

The gene encoding human mitochondrial malate dehydrogenase (hMDH2) was amplified by polymerase chain reaction (PCR), including the *Nde*I and *EcoR*I endonuclease sites. The PCR product was cloned into the *Nde*I and *EcoR*I sites of the pET-28a expression vector (Merck KGaA, Darmstadt, Germany). Recombinant hMDH2 includes the hMDH2 sequences, as well as the 6 histidine-tag (6His) with a thrombin protease cleavage site at its N-terminus. The construct was transformed into *E. coli* Rosetta2 (DE3) (Merck KGaA, Darmstadt, Germany) for expression. Cells were grown at 37 °C until OD_600_ of culture reached to 0.7–0.8 and induced with 0.2 mM isopropyl-β-D-thiogalactopyranoside (IPTG) (Merck KGaA, Darmstadt, Germany) at 18 °C for 20 h. The harvested cells were re-suspended in 25 mM Tris-HCl, pH 7.2, 500 mM NaCl, 30 mM imidazole with the addition of phenylmethylsulfonyl fluoride (PMSF) powder and disrupted by sonication on ice. Cell debris was pelleted by centrifugation, and the supernatant was loaded on an open column filled with cOmplete His-Tag purification resin nickel-NTA resin (Roche, Penzberg, Germany) and for further purification, size-exclusion chromatography was performed using HiLoad 16/600 Superdex 75 pg column (GE Healthcare, Chicago, IL, USA), equilibrated with 25 mM Tris-HCl, pH 7.2, 100 mM NaCl and 1 mM tris(2-carboxyethyl)phosphine (TCEP) (Goldbio, MO, USA). The eluents from the size-exclusion chromatography were concentrated by Amicon Ultra (Merck KGaA, Darmstadt, Germany) to 15 mg/mL. The protein was stored at −80 °C. The protein concentration was determined by measuring the UV absorbance at 280 nm with the molar extinction coefficient of a monomeric hMDH2, 7450.

### 2.2. Crystallization and Data Collection

Initial crystal screenings were performed with crystallization screen kits (MCSG I and II, Anatrace, Maumee, OH, USA) via the sitting drop vapor diffusion method at 15 °C. The hMDH2 native crystals were grown under the condition of a solution containing 0.1 M Na_2_HPO_4_;citric acid, pH 4.2, 40% (*v/v*) polyethylene glycol (PEG) 300. The crystallization condition was optimized by the addition of 10% dimethyl sulfoxide (DMSO) using the hanging drop vapor diffusion method.

For the complex structures, the substrates and cofactors of hMDH2 were soaked into the initial crystals. Since the initial crystal contained the citric acid, the crystals were transferred to a solution containing 0.1 M Na_2_HPO_4_;HCl, pH 4.2, 40% (*v/v*) PEG 300, 10% DMSO without citric acid, and incubated for 2 h. These crystals were used for the ligand soaking. To obtain different combinations of the ligand bound hMDH2 crystals, 5 mM β-nicotinamide adenine dinucleotide hydrate (NAD, Biobasic, Markham, ON, Canada), 5 mM _L_-(-) malic acid (Sigma-aldrich, St-Louis, MO, USA), 5 mM β-nicotinamide adenine dinucleotide, reduced disodium salt hydrate (NADH, Sigma-aldrich, St-Louis, MO, USA), and 5 mM oxaloacetic acid (Sigma-aldrich, St-Louis, MO, USA) were added to the crystallization solution, respectively or together. Diffraction data were collected on beam line BL7A of PAL/PLS (Pohang, Korea) and BL44XU of SPring-8 (Hyogo, Japan).

### 2.3. Structure Determination and Refinement

Diffraction data on hMDH2 were processed using the *HKL2000* program suite [16]. All further data and model manipulations were carried out using the *CCP4* program suite [17]. Phases for all the hMDH2 structures were solved by molecular replacement with *Phaser* [18], using the hMDH2 (PDB entry, 2DFD) structure as a template model. The resulting models were further refined using *REFMAC5* [19] to create the initial electron-density maps. Electron-density maps were calculated and the structures were coordinated to give the best fit to both the *2F_o_ − F_c_* and *F_o_ − F_c_* maps. Maximum-likelihood refinement was performed using *REFMAC5* after model building was performed in *Coot* [20]. The dictionary definitions for the ligands were created using *PRODRG* [21]. Statistics for the data processing and refinement of the final models are summarized in Table 1. After refinement, the quality of the models was validated using *MolProbity* [22]. The quaternary protein structure analyses were performed via *PDBePISA* [23]. Structure representations were created with using the *PyMOL* molecular-graphics program (The PyMOL Molecular Graphics System, Schrödinger, LLC., New York, NY, USA) [24]. The coordinates of the all six refined structures were deposited in the Protein Data Bank as entries 4WLN, 4WLE, 4WLF, 4WLO, 4WLU, and 4WLV for the phosphate, citrate, _L_-malate, oxaloacetate/NADH, _L_-malate/NAD, and NAD bound structures, respectively.

### 2.4. Enzymatic Assay

The enzyme activity of hMDH2 was conducted by NADH oxidation assays, where the oxidation of NADH was measured by monitoring the absorbance at 340 nm. The reaction was performed in 100 mM potassium phosphate buffer, pH 7.5, with 0.15 nM hMDH2, 0.25 mM oxaloacetic acid, and 0.13 mM NADH. To check the effect of phosphate on the enzymatic activity of hMDH2, HEPES and Tris-HCl buffers were also used The concentrations of the buffer components were 20, 50, and 100 mM for phosphate and Tris-HCl buffers, and 20 and 50 μM for HEPES buffer.

### 2.5. ITC Measurement

ITC measurements were performed at 25 °C with a Nano ITC (TA Instruments, New Castle, DE, USA). The hMDH2 was prepared in a solution containing 25 mM Tris-HCl, pH 7.2, 100 mM NaCl, and 1 mM TCEP, and the cofactors were dissolved in the same buffer. For the NADH titration, 30 μM hMDH2 was placed in the sample cell and the 230 μM NADH was loaded into the syringe. A single injection of 2.5 μL was made at every 200 s for 20 injections. A competitive binding assay was carried out for NAD binding. Pre-incubated 30 μM hMDH2 and 600 μM NAD was filled in the sample cell, and 230 μM NADH was titrated in the same manner. Blank titration was performed under the same conditions, without protein, in the sample cell, and the background heat was subtracted from the integrated data. Data analyses were performed using the NanoAnalyze software (TA Instruments, New Castle, DE, USA) with an independent model for NADH and a competitive replacement model for NAD.

## 3. Results

### 3.1. Overall Structure of hMDH2

The hMDH2 was expressed in *E.coli* and purified by Ni-NTA affinity chromatography and size exclusion chromatography. The final purity of hMDH2 was more than 95%, as judged by SDS-PAGE (Figure S1). In the initial crystal screening, hMDH2 crystals were produced from the solutions containing the citrate or malate. From those conditions, small crystals were grown in a solution containing 0.1 M Na_2_HPO_4_;citric acid, pH 4.2, 40% (*v/v*) PEG 300. Under the presence of 10% DMSO, larger crystals were obtained (Figure S1). The optimized crystals were used for ligand soaking.

**Figure 1:**
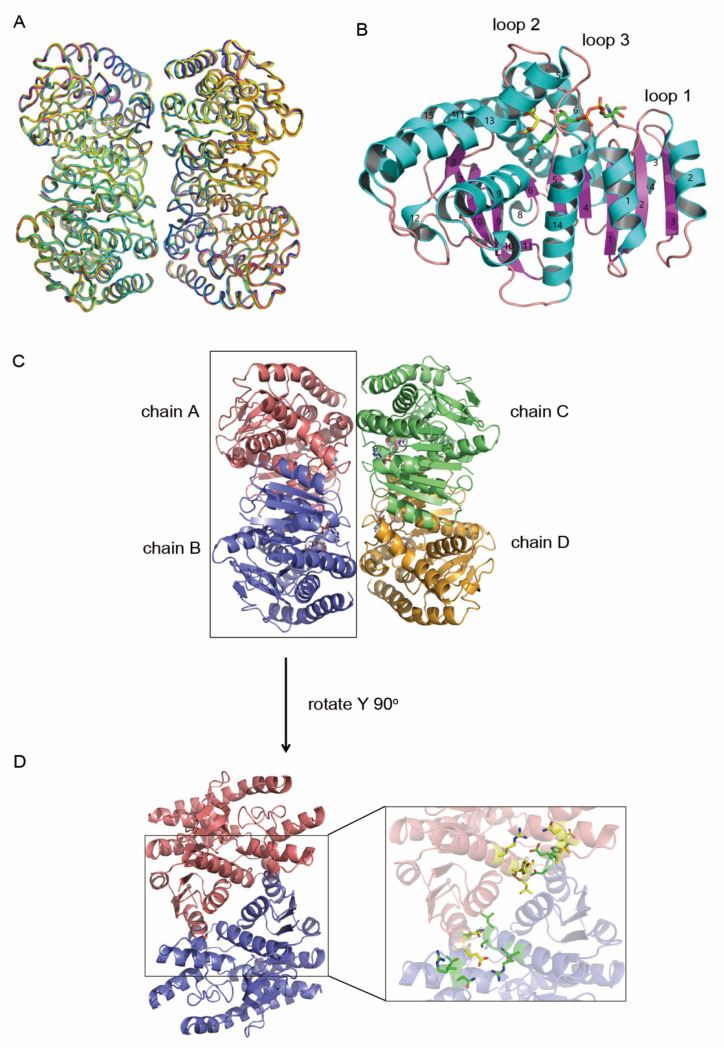
Overall structure of hMDH2. (**A**) Ribbon representations of the superimposed structures of hMDH2 complexed with various ligands (citrate bound—green; _L_-malate and NAD bound—cyan; _L_-malate bound—magenta; NAD bound—yellow; PO_4_^−^ bound—blue; oxaloacetate and NAD bound—orange). (**B**) Cartoon diagram of hMDH2 monomer with _L_-malate and NAD. The α-helices are shown in cyan, the β-strands in magenta and the loops in pink. The _L_-malate and NAD are shown as sticks colored with yellow and green, respectively. The α-helices and β-strands are numbered in the order of appearance from the N-terminus, respectively. Three loops surrounding the active site are indicated. (**C**) A cartoon diagram showing the overall conformation of the hMDH2 tetramer from the _L_-malate and NAD complex structure. Bound _L_-malate and NAD molecules are shown in ball-and-stick representations. (**D**) Subunit A and B of hMDH2 are shown as a cartoon representation by 90° rotation around the *Y*-axis from (**C**). The dimeric interface is enlarged in a box. Residues involved in the hydrogen bond interactions in the dimeric interfaces are shown as yellow sticks for chain A and green sticks for chain B. The residues are not indicated, for the clarity of the figure (please see Table S1).

To describe the structural basis for the catalytic mechanism of hMDH2, several types of complex structures of hMDH2 were solved based on the crystal soaking method. The six different structures of hMDH2 with (1) citrate, (2) _L_-malate, (3) NAD, (4) _L_-malate and NAD, (5) oxaloacetate and NADH, and (6) phosphate were determined respectively. The chemical structures of citrate, _L_-malate, oxaloacetate, NAD, and NADH are shown in Figure S2. The overall structures were similar to each other (Figure 1A). The electron densities of N-terminal residues (the expression tag and residues from 20 to 23) and a C-terminal residue (338) were not visible in any of the six structures, probably owing to structure disorder.

The overall structure of hMDH2 was similar to other MDHs having an N-terminal NAD-binding domain and a C-terminal catalytic domain. Each subunit contained 15 α-helices and 11 β-strands (Figure 1B). The NAD-binding domain represented a classical Rossmann fold composed of six parallel β-strands (β1–β6) connected by α-helices and loops. All the crystal structures of hMDH2 were tetramers assembled as a dimer of dimers (Figure 1C). The subunits A and B formed one dimer, and subunits C and D formed another. The dimeric interface was primarily stabilized by helix–helix interactions in which the six α-helices (α1, α2, α9, α10, α13, and α14) were involved. More than 10 residues (Asp67, His70, Glu72, Arg176, Asn178, Thr179, Lys241, Thr248, Leu249, Ser250, Tyr253, and Arg257) participated in hydrogen bond interactions (Figure 1D and Table S1), and a wide range of hydrophobic interactions were also responsible for the dimerization. The buried surface area between A and B (C and D) was approximately 1550 Å^2^ per monomer, which was roughly 13% of the total surface area of a monomer. In contrast, the buried surface areas of A–D and B–C were about 440 Å^2^, which was about three times less than A–B and C–D. The buried surface area of all six determined structures is listed in Table S2. It has been well known that most MDHs are stable and active as dimers [7]. In addition, the MDH from *E. coli* loses specific activity in the monomer state, implying that the catalytic mechanism relies upon the dimeric structure [25]. When the structure of hMDH2 in complex with _L_-malate and NAD was compared to the previously reported protein with the same ligands, 2DFD in PDB, the root mean square deviation (r.m.s.d.) value of the backbone Cα was 0.235 Å and the positions of _L_-malate and NAD were almost identical.

### 3.2. Obtaining Ligand Bound Structures

In the first crystallization step, the citrate bound hMDH2 structure was obtained because the citrate was included in the crystallization condition. The citrate occupied the substrate binding site in the refined structure. The *E. coli* MDH structure [13] also possessed the citrate in the active site, for the same reason mentioned above. To produce the apo-crystal, the crystallization of hMDH2 was tried under the citrate-free condition, but no crystal was obtained. Thus, the crystals containing citrate were used for soaking the substrate and cofactor to produce the complex crystals; however, citrate at the substrate binding site hampered the production of the ligand bound crystals. This problem was solved by transferring the crystals to a citrate-free crystallization solution. Structural analysis of this citrate-free crystal revealed that citrate was not present in the active site; however, it was replaced by three phosphate ions. Thereafter, these citrate-free crystals were soaked into the various combinations of substrate and cofactor containing solutions. Consequently, different ligand-bound structures have been determined, which accounted for the binding mode of different ligands.

### 3.3. Comparison of _L_-Malate and Citrate Binding

**Figure 2:**
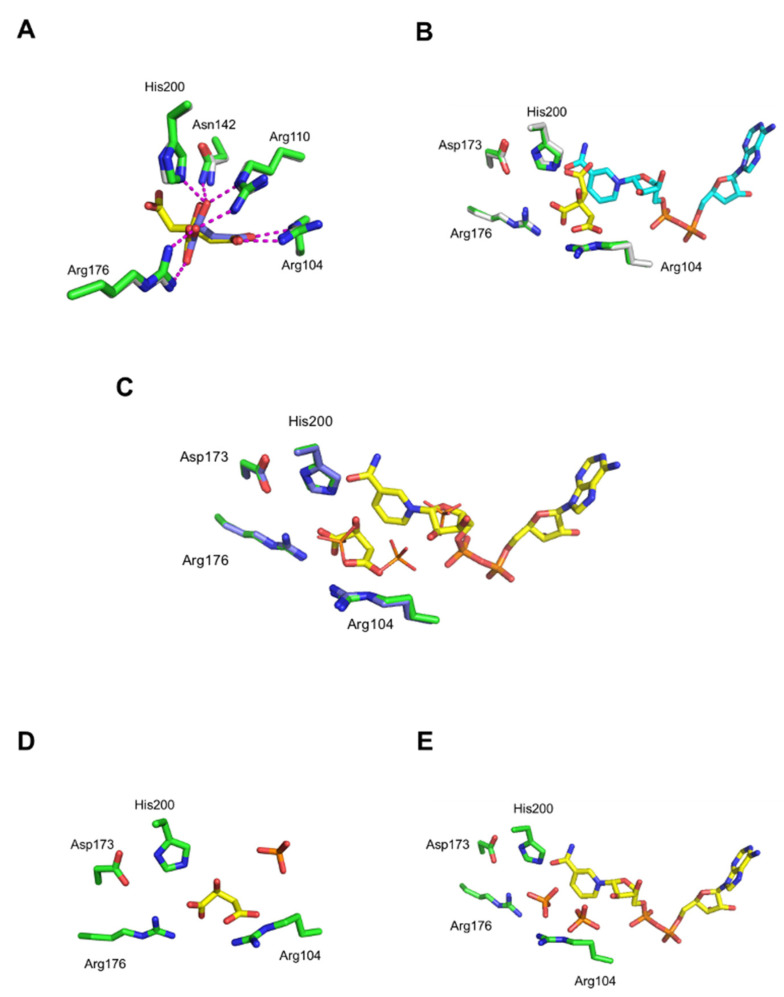
Comparison of active site of hMDH2. The active site is shown, and the residues and ligands are displayed as sticks or lines. (**A**) Superimposed active sites of citrate bound (residues in green and citrate in yellow) and _L_-malate bound (residues in gray and _L_-malate in purple) structures. (**B**) Superimposition of citrate bound (colors the same as in A) and NAD bound (residues in white and NAD in cyan) structures. (**C**) Superimposition of phosphate bound (residues in purple and phosphate in orange) and _L_-malate-NAD bound (residues in green and ligands in yellow) structures. Phosphates are displayed as lines. (**D**) Active site of the malate bound structure; (**E**) active site of the NAD bound structure. In both (**D**,**E**), phosphates are colored in orange.

Multiple sequence alignment exhibited the highly conserved residues of MDHs throughout the various species (Figure S3) [26]. All MDHs possess a histidine residue (His200 in hMDH2) coupled with an aspartate (Asp173 in hMDH2) that shuttles protons in the active site. In this study, the _L_-malate bound structure was ascertained and compared with the previously determined citrate bound form. Both _L_-malate and citrate bound in the same site of hMDH2 and formed hydrogen bonds with the surrounding residues, such as Arg104, Arg110, Asn142, Arg176, and His200 (Figure 2A). Three arginine residues were critical to accommodate the carboxylate groups in the substrate binding site. Citrate has three carboxylate groups, and two of those bound to three arginine residues via hydrogen bonds, but the third carboxylate did not interact with any hMDH2 residues involved in _L_-malate binding. The citrate binding to hMDH2 was similar to the pattern of citrate binding in the *S. scrofa* MDH structure [27]. The third carboxylate occupied the same position for the nicotinamide ring of NAD binding (Figure 2B). In the superimposed structures of the citrate bound and NAD bound hMDH2, the third carboxylate of the citrate mentioned above and the nicotinamide of NAD positioned at the same site. Because of the steric clash between one carboxylate of citrate and the nicotinamide of NAD or NADH, citrate hampers the binding of the cofactors to hMDH2 and inhibits the enzymatic activity of hMDHs. On the other hand, there was enough space between _L_-malate and NAD and no structural clash occured (Figure 2C). If _D_-malate, the enantiomer of _L_-malate, was introduced to the substrate binding site with the same hydrogen bonds with the sidechain of Arg176, a structural clash between the carboxylic or hydroxyl group of _D_-malate and nicotinamide of NAD would occur (Figure S4). This suggests the mechanism of the substrate specificity of this enzyme between the enantiomers of malate.

### 3.4. Comparison of Active Site

Three phosphate ions were found in the absence of both a substrate and a cofactor. To examine the binding site of phosphate, the phosphate bound structure was superimposed with _L_-malate and/or NAD bound structures. In the superimposed structures, two phosphate ions were located in the substrate binding site and a third in the position of the ribose ring close to nicotinamide moiety of NAD (Figure 2C). The phosphate ion was not found in the _L_-malate and NAD bound form, whereas one phosphate was found in an empty NAD binding site in the _L_-malate bound structure. Two phosphate ions were found in the substrate binding site in the NAD bound structure. (Figure 2D,E). Interestingly, the enzymatic activities of hMDH2 were different when the assays were performed in the buffer with and without phosphate ions (Figure S5). In the phosphate buffer, hMDH2 was active; hence, the oxidation of NADH to NAD was detected by measuring the absorbance at 340 nm, where NAD does not absorb the light. However, in the phosphate-free conditions, the catalysis occurred very slowly in Tris-HCl buffer, or negligibly in HEPES buffer. When phosphate was added to the reaction solution with the HEPES or Tris-HCl buffers, the rate of reaction was increased (Figure S5). Thus, the phosphate binding to substrate and cofactor binding sites seems to affect the enzyme activity. In other crystal structures of MDHs, anion bound forms were often observed because the active site contained a large number of positive residues.

The _L_-malate binding to hMDH2 was not changed in the presence of NAD. The active site structures with _L_-malate in the presence and absence of NAD (Figure 2C,D, respectively, were almost identical to each other. We suppose that the same pattern of binding was retained for the oxaloacetate and NADH cases, even though direct evidence was not presented, since the oxaloacetate bound hMDH2 was not able to be determined in our work.

The substrate and cofactor bound structures displayed that three loops formed a binding groove (Figure 1B). Many hydrogen bonds were formed between the enzyme and the ligands and the length of hydrogen bonds were summarized for each structure (Table S3). The ribose and pyrophosphate of NAD formed hydrogen bonds with Asp57, Asn117, Asn142 and Gly35, Ile36 of hMDH2, respectively. The nicotinamide interacted with Ile140 and Val169 via hydrogen bonds which seemed to be important in the catalytic activity. The lengths of hydrogen bonds formed between nicotinamide and Ile140 and Val169, respectively, in _L_-malate and NAD bound structure were shorter than those in hMDH2 structure with NAD.

### 3.5. NAD, _L_-Malate vs. NADH, Oxaloacetate

**Figure 3:**
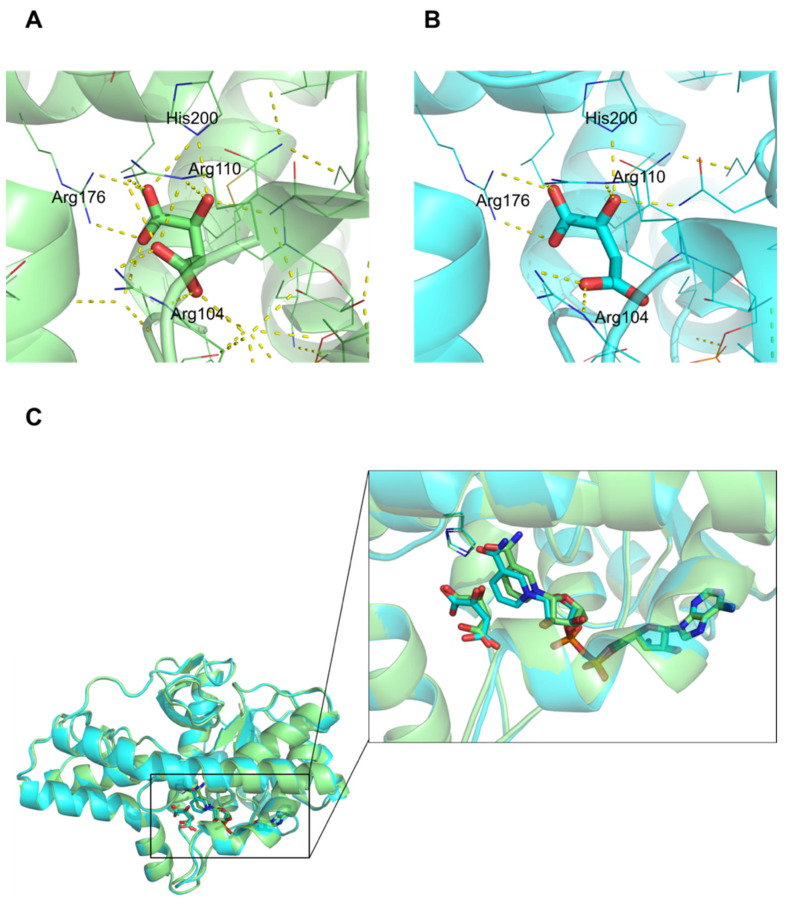
Comparison of _L_-malate-NAD bound form (**A**) with oxaloacetate-NADH bound form (**B**). Substrates are shown as sticks, and interacting residues are shown as lines (_L_-malate binding in lime; oxaloacetate binding in cyan). (**C**) Cartoon diagram of hMDH2 complexed with substrates and cofactors. The active site is enlarged. Substrates and cofactors are shown as sticks, and His200 residue is represented as a line (colors are the same as in (**A**,**B**)).

In the oxaloacetate and NADH bound structure, the length of the hydrogen bonds involved in His200, which is one of the key residues in proton transfer, was slightly longer than that in the _L_-malate and NAD bound structure (Table S3). Three arginine residues, Arg104, Arg110, and Arg176, along with His200, had interactions via hydrogen bonds with all oxygen atoms of _L_-malate (Figure 3A). Arg110, Arg176, and His200 retained the similar interaction as oxaloacetate; however, Arg104 paired with only one oxygen atom of oxaloacetate (Figure 3B). The O1 atom of oxaloacetate did not form a hydrogen bond with any residues of MDH2. NAD and NADH bound to hMDH2 in a similar pattern, except for the nicotinamide moiety. The positions of the nicotinamide ring were different, where the ring was pulled towards the center of the active site and tilted by 16~17° in the NADH bound form (Figure 3C).

### 3.6. Cofactor Binding Affinity

**Figure 4:**
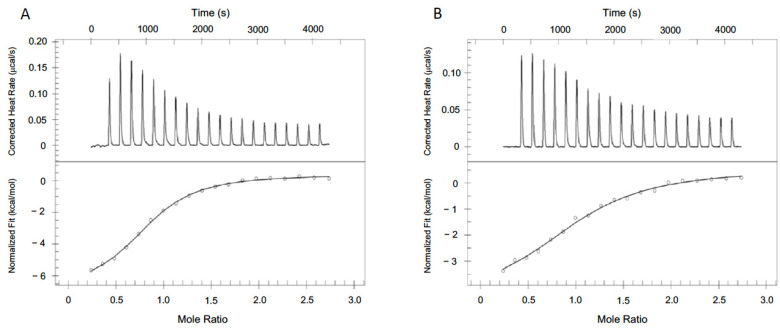
The measurements of the binding affinity of the cofactors to hMDH2. (**A**) Binding affinity measurement of NADH and hMDH2 by ITC. The upper panel shows the heat changes for injection of 2.5 μL aliquots of 230 μM NADH into 30 μM hMDH2. The lower panel shows the integrated heats from the raw data. The data were fitted with an independent model. (**B**) Binding affinity measurement of NAD and hMDH2 by the competitive replacement method of ITC. The upper panel shows the heat changes for the injection of 2.5 μL aliquots of 230 μM NADH into 30 μM hMDH2 pre-incubated with 600 μM NAD. The lower panel shows the integrated heats from the raw data. The data were fitted with a competitive replacement model provided by TA Instruments.

ITC experiments were conducted to measure the binding affinities of the cofactors. The binding of NADH to hMDH2 was endothermic, and the heat was released upon titration. The NADH titration data were fitted by applying the independent model (Table 2, Figure 4A). The N value of the fitting was 0.83; thus, the binding stoichiometry between hMDH2 and NADH seemed to be 1 to 1. The change in binding free energy (ΔG) was −7.52 kcal/mol and the dissociation constant (*K_d_*) was 3.05 μM, which is very similar to the previously reported value, 3.8 ± 0.2 μM, obtained with porcine mitochondrial MDH [28]. The binding of NADH to hMDH2 was mostly enthalpy-driven (ΔH, −7.29 kcal/mol), with minor favorable entropic contribution (-TΔS, −0.23 kcal/mol) at 298.15 K.

On the contrary, the direct titration of NAD to hMDH2 released too little heat to determine the thermodynamics; thus, a competitive replacement experiment was performed. To characterize the thermodynamics of NAD and hMDH2 binding, the pre-incubated protein with NAD was placed in the cell and aliquots of NADH were injected into the protein. The NAD titration data were fitted via a competitive replacement model (Figure 4B). To fit the NAD titration data, the previously obtained NADH thermodynamic parameters were used. The changes of binding free energy (ΔG), enthalpy (ΔH), and entropy (-TΔS) were −4.59, −3.65, and −0.94 kcal/mol, respectively, and the *K_d_* value for NAD to hMDH2 was 434 μM, under the assumption of 1 to 1 binding. NADH bound about 660-fold stronger to hMDH2 than did the NAD.

## 4. Discussion

Most studies on MDH, including active site conformation and catalytic mechanism, have been conducted using the *E.coli* enzyme. The sequence identity and similarity between *E. coli* MDH and human MDH are 59% and 71%, respectively. Greater similarities are found between the functionally significant residues, especially in the active site. In addition, the three-dimensional structures of hMDH2 are very similar to those of MDH from various species, including *E. coli* MDH.

In order to investigate the difference in the binding modes among the natural ligands, the complex structures were solved in various combinations. To obtain the hMDH2 crystal, initial screening was conducted under approximately 400 conditions, and hMDH2 crystals were produced in several conditions. All the crystalized conditions contained citrate, which agrees well with the previous structural studies. No crystals were formed without substrate or substrate mimics. Even though the citrate ion seems to play an important role in MDHs crystal packing, citrate occupied the active site and interfered with the binding of natural substrates and cofactors. This effect could be solved by placing crystals under citrate-free conditions before soaking the desired natural ligands. The NAD and NADH, which are larger in size than the substrates, went through a solvent channel to their binding sites during the soaking period, which made it possible to solve the cofactor bound complex structures. In this way we have determined five combinations of substrate and cofactor bound hMDH2 structures and one structure with phosphate in the active site. The omit *F_o_* *− F_c_* electron density maps of hMDH2, with different substrates and cofactors, are presented in Figure S6. The overall structures of all six structures were very similar to each other and to the previous homologues, and the r.m.s.d. values of backbone Cα of six complexes were within 0.3 Å.

**Figure 5:**
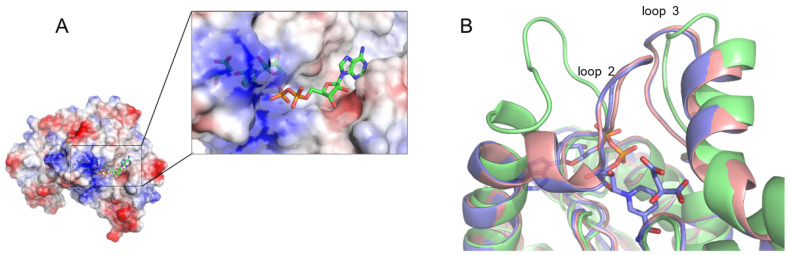
The closed conformation of the active site (**A**). The monomer structure of the _L_-malate and NAD bound hMDH2 is shown in electrostatic potential surface representations, and _L_-malate and NAD are displayed as sticks. The active site is enlarged in a box. The nicotinamide moiety and _L_-malate are bound in a positively charged pocket. The comparison of the active loop (**B**). Cartoon diagram of the active site is shown. _L_-malate and NAD are displayed as sticks (*E. coli* apo-structure); PBD code, 3HHP—lime; _L_-malate and NAD bound hMDH2—light purple; phosphate bound hMDH2—salmon).

Regardless of types of ligands, all complex structures containing the substrate, substrate analogue, cofactors, or anion showed the loop-closed conformations. The active loop (loop 2 in Figure 1B), including Arg104 and Arg110 residues, has been shown to play a pivotal role in catalysis. In a loop-closed conformation, the bound substrate and the nicotinamide moiety of the cofactors were completely blocked from the exterior by this loop (Figure 5A), and the conversion of _L_-malate to oxaloacetate would be facilitated via the proton transfer coupled with the reduction of NAD to NADH. Unfortunately, the crystal structure in which the active site is located in an open conformation, was not obtained in our work. To examine the difference between the open and closed conformation of MDH, the active site of hMDH2 with the substrate and cofactor was compared to that of the *E.coli* MDH apo-structure, which had a loop-open conformation (Figure 5B) [29]. In the loop-open conformation, the substrate binding site is almost exposed to the solvent, which facilitates the easy access of substrates and cofactors to the active site. Once the binding occurs, the loop undergoes the conformational change to a closed state. Since the residues surrounding the active sites are very hydrophilic, the anionic molecules, such as phosphate ions or citrate (even though these are not the substrate of MDHs), bind to the active site via the hydrogen-bond network and bring about the conformational change of the enzyme. At this stage, the key residues in the substrate binding site, Arg104 and Arg110, determine whether the bound molecule is the genuine substrate or not. If citrate bound to the active site, it would inhibit the enzymatic activity of the MDHs, and if phosphate bound to the active site, the natural substrate would replace the phosphate, and the enzymatic reaction would be initiated. We suppose that the loop-closed conformation is an enzymatically active state, and the phosphate ion, which is abundant and ubiquitous in any organism, is very important in maintaining the active conformation and subsequent enzymatic activity of MDHs. Under the phosphate free solutions, hMDH2 would stay in an open-conformation and remain enzymatically inactive; therefore, the activity of hMDH2 in HEPES or Tris-HCl buffers was negligible (Figure S5).

The binding of cofactors to hMDH2 was characterized by ITC experiments. In ITC, the heat released or absorbed along the titration corresponds to the fraction of the bound ligand. When a small aliquot of NADH solution was injected into the hMDH2 solution, most of the added NADH bound to hMDH2, and the ITC instrument measured the heat change by integrating the peak area (Figure 4A). During the following titrations of NADH to hMDH2, the released heat gradually decreased because the binding sites of NADH on hMDH2 were saturated. Increased NADH concentrations led to the saturation of hMDH2 and finally, to less heat being released. We performed ITC experiments under the phosphate free condition in Tris-HCl buffer. Indeed, we tried various combinations of titration experiments in varieties of buffer conditions, but it was impossible to obtain meaningful data to characterize the binding thermodynamics of hMDH2. The thermodynamics of NADH binding to hMDH2 were characterized, and they implied that the binding was 1:1 with the *K_d_* of 3.05 μM. The binding was driven by a favorable enthalpic process, with a minor entropic contribution. On the other hand, the binding of NAD to hMDH2 was much weaker than that of NADH, with *K_d_* in millimolar range, but the binding of NAD to hMDH2 was also both enthalpy- and entropy-favorable. The enthalpic contribution in both NADH and NAD binding to hMDH2 should be attributed mainly to the formation of many hydrogen bonds, as was shown in the structural data. However, there is a striking difference in the enthalpy changes: −3.45 kcal/mol between the binding of NADH and NAD to hMDH2. The value is quite large regarding similar substrate binding because NADH is the reduced form of NAD. The structural differences of NADH and NAD in hMDH2 complexes were very little, and the orientation of the nicotinamide ring of NADH was slightly tilted, compared to that of NAD. It is unclear whether the tilting of the nicotinamide ring significantly contribute to binding. Even though the binding affinity of NAD to hMDH2 is very low, the continuous supply of _L_-malate from fumarate by fumarase, and the consumption of oxaloacetate to produce citate by citrate synthase in TCA cycle drive the conversion of malate to oxaloacetate, with the reduction in NAD to NADH, which process is thermodynamically unfavorable in a standard condition.

## 5. Conclusions

In this work, we have produced six complex structures of hMDH2, with combinations of substrates and cofactors, and with a phosphate ion. The production of the complex crystal was enabled by transferring the citrate-containing hMDH2 crystal to a citrate-free buffer before soaking. The structure of hMDH2 was almost identical, regardless of the bound ligands, and in the loop-closed conformation, even in the phosphate bound form. The structural comparison between this work and an open conformation of *E.coli* MDH, an in vitro enzymatic assay, and ITC experiments prove the significance of the phosphate ion in the maintenance of an active, loop-closed conformation of hMDH2 and in its catalytic mechanism.

## Figures and Tables

**Table 1:** Data collection and refinement statistics.

	MDH2_CIT ^a^	MDH2_PO_4_	MDH2_LMR ^a^	MDH2_NAD	MDH2_LMR, NAD	MDH2_OAA ^a^, NAI ^a^
PDB ID	4WLE	4WLN	4WLF	4WLV	4WLU	4WLO
Data collection						
Space group	p212121	p212121	p212121	p212121	p212121	p212121
Cell dimensions						
a, b, c (Å)	59.70, 151.22, 154.65	59.96, 151.94, 155.05	60.25, 152.18, 155.03	58.86, 152.02, 155.67	59.98, 152.14, 155.04	59.99, 152.11, 155.87
α, β, γ (°)	90, 90, 90	90, 90, 90	90, 90, 90	90, 90, 90	90, 90, 90	90, 90, 90
Resolution (Å) *	45.10-1.90 (1.93-1.90)	32.56-2.28 (2.32-2.28)	34.86-2.20 (2.24-2.20)	30.86-2.40 (2.44-2.40)	30.79-2.14 (2.19-2.15)	28.35-2.50 (2.54-2.50)
R_merge_ ^b^	9.2 (80.9)	13.1 (58.1)	6.8 (33.3)	7.9 (46.1)	7.4 (38.7)	7.5 (41.8)
I/σ_I_	31.3 (3.3)	34.04 (6.0)	54.0 (10.0)	51.2 (9.08)	55.5 (10.5)	47.8 (7.6)
Completeness (%) *	99.8 (99.9)	99.8 (100.0)	95.9 (91.6)	99.9 (100.0)	99.8 (100.0)	99.0 (98.9)
Redundancy *	7.1 (6.8)	14.2 (4.7)	11.1 (10.2)	14.3 (14.3)	13.6 (13.5)	12.8 (11.4)
Refinement						
Resolution (Å) *	45.10-1.90 (1.93-1.90)	32.56-2.28 (2.32-2.28)	34.86-2.20 (2.24-2.20)	30.86-2.40 (2.44-2.40)	30.79-2.14 (2.19-2.15)	28.35-2.50 (2.54-2.50)
No. reflections	104,532	62,209	66,329	53,618	74,182	47,440
R_work_/R_free_ (%) ^c^	21.8/23.8	21.7/24.6	22.7/24.7	20.8/24.8	21.6/24.4	21.1/25.5
No. atoms	9417	9430	9351	9514	9562	9488
protein	9258	9248	9252	9248	9248	9251
ligand	52 (CIT: 52)	60 (PO_4_: 60)	56 (_L_-MLT: 36, PO_4_: 20)	216 (NAD: 176, PO4: 40)	212 (_L_-MLT: 36, NAD: 176)	212 (NADH: 176, OAA: 36)
water	107	122	43	50	102	25
Mean B-factors (Å^2^)	35.047	33.354	37.71	38.97	35.08	45.593
protein	35.37	33.68	38.10	39.31	35.43	45.91
ligand	36.93 (CIT: 36.93)	34.21 (PO_4_: 34.21)	_L_-MLT: 37.52, PO_4_: 54.87	NAD: 37.32, PO_4_: 54.76	_L_-MLT: 35.06, NAD: 34.80	NADH: 54.55, OAA: 50.81
water	31.73	30.53	35.50	30.82	31.88	35.68
R.m.s. deviations						
Bond lengths (Å)	0.004	0.005	0.005	0.005	0.005	0.006
Bond angle (°)	0.898	1.226	1.026	1.190	0.984	1.066

^a^ The names of ligands followed the abbreviations in the PDB. CIT stands for citrate; LMR for _L_-malate; OAA for oxaloacetate; and NAI for NADH. * Values in the parentheses refer to the highest resolution shells. ^b^ *R*_merge_ = Σ*_i_*Σ*_i_*|*I*(*h*)*_i_* − <*I*(*h*)>|/Σ*_h_*Σ*_i_I*(*h*)*_i_*, where *I*(*h*) is the intensity of reflection *h*, Σ*_h_* is the sum of all reflections, and Σ*_i_* is the sum of the *i* measurements of reflection *h*. ^c^ *R*_work_ = Σ||*F*_obs_| − |*F*_calc_||/Σ|*F*_obs_|; 5% of the data was set aside for *R*_free_ calculation.

**Table 2:** Data collection and refinement statistics at 298.15 K.

Cofactor	ΔH (kcal mol^−1^)	TΔS (kcal mol^−1^)	ΔG (kcal mol^−1^)	N	K_d_ (μM)
NADH	−7.29 ± 0.43	−0.23	−7.52	0.83 ± 0.06	3.05 ± 0.06
NAD	−3.65 ± 3.20	−0.94	−4.59	N.A. ^a^	2306 ± 1733

^a^ In a competitive replacement, the stoichiometry of 1 to 1 was assumed; thus, the N value is not available from the fitting.

## Data Availability

Not applicable.

## Supplementary Materials

The following supporting information can be downloaded at: https://www.mdpi.com/article/10.3390/biom12091175/s1. Figure S1: Purified hMDHs after size-exclusion chromatography; Figure S2: Chemical structures of ligand, substrates, and cofactors of hMDHs; Figure S3: Multiple sequence alignment of hMDH2 and its homologues; Figure S4: Suggested the mechanism of the substrate specificity; Figure S5: Enzymatic activity of hMDH2 in phosphate, HEPES, and Tric-HCl buffers; Figure S6: Composite (2 *F_o_ − F_c_*, gray, contoured at 1σ) and difference (*F_o_ − F_c_*, blue/red, contoured at 3σ) electron density maps of hMDH2; Table S1: Hydrogen bond interactions across the dimeric interface; Table S2: Surface area comparisons of different ligands bound to hMDH2 structures; Table S3: Hydrogen bond interactions of hMDH2 and ligands.
