# Supplementary material for: Structural Comparison of hMDH2 Complexed with Natural Substrates and Cofactors: The Importance of Phosphate Binding for Active Conformation and Catalysis
Source: Biomolecules. 2022 Aug 25;12(9):1175. doi: 10.3390/biom12091175 (PMC9496400; doi:10.3390/biom12091175)
Supplement: Supplementary file 1 [file biomolecules-12-01175-s001.zip › biomolecules-1862087-supplementary.pdf]

Supplementary figures and tables for

Structural comparison of hMDH2 complexed with natural substrates and cofactors: Importance phosphate binding for active conformation and catalysis

Yumi Eo [1], Men Thi Hoai Duong [1] and Hee-Chul Ahn [1,*]

[1] College of Pharmacy, Dongguk Univeristy-Seoul, Goyang, Gyeonggi 13026, Republic of Korea
* Correspondence: hcahn@dongguk.edu; Tel.: +82-31-961-5221

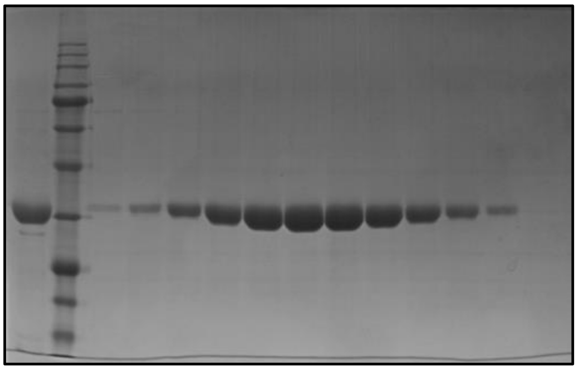

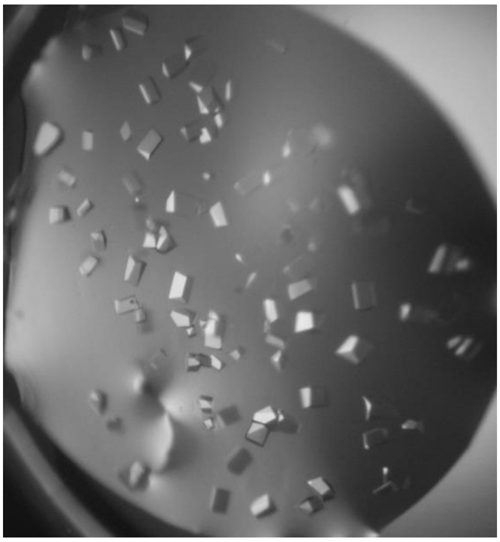

Figure S1. Purified hMDHs after size-exclusion chromatography (top). Native crystals of hMDH2 (bottom). MDH2 native crystals were produced in a solution containing 0.1M Na_2HPO_4;citric acid, pH 4.2, 40% (v/v) PEG 300. For the optimization 10% DMSO was added to the crystallization condition.

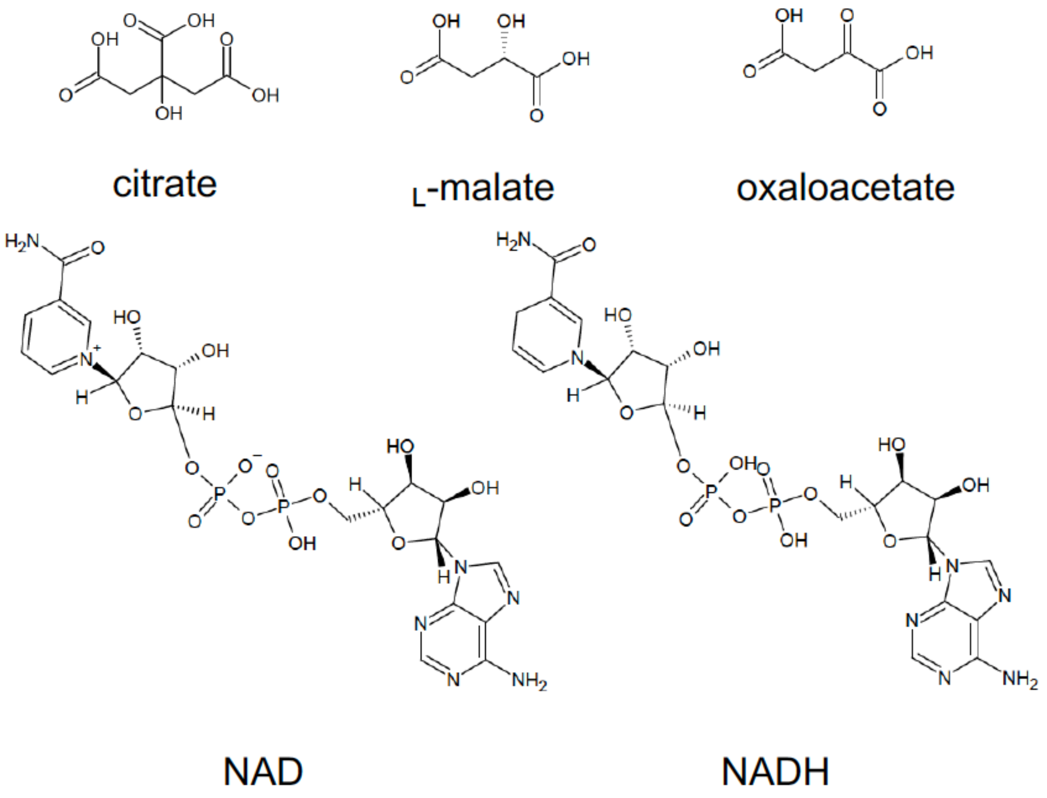

Figure S2. Chemical structures of ligand, substrates, and cofactors of hMDHs.

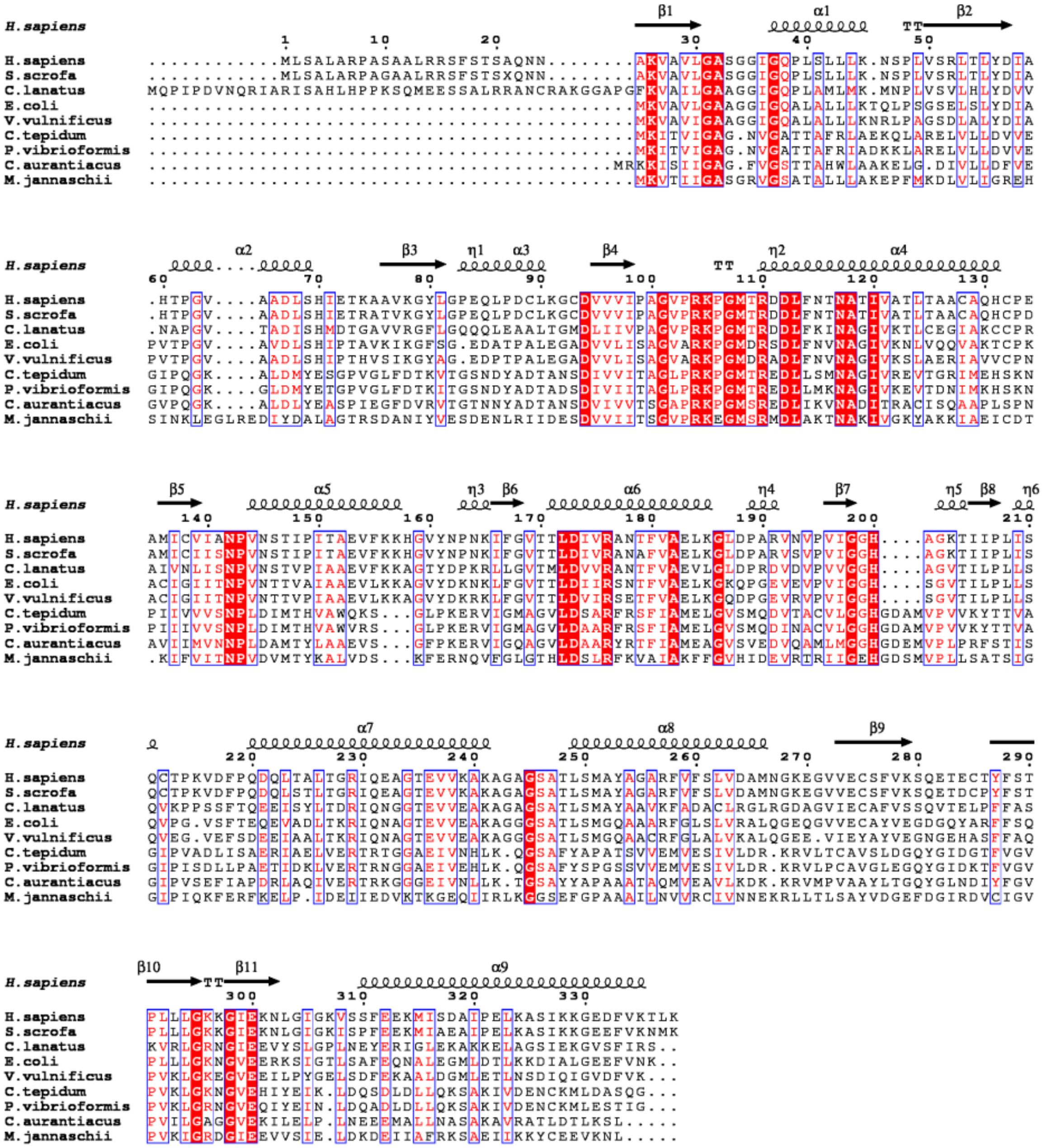

Figure S3. Multiple sequence alignment of hMDH2 and its homologues. The completely conserved residues are framed with red background. The highly conserved residues are in blue boxes with red letters. The secondary structures of MDH are represented on the top row.

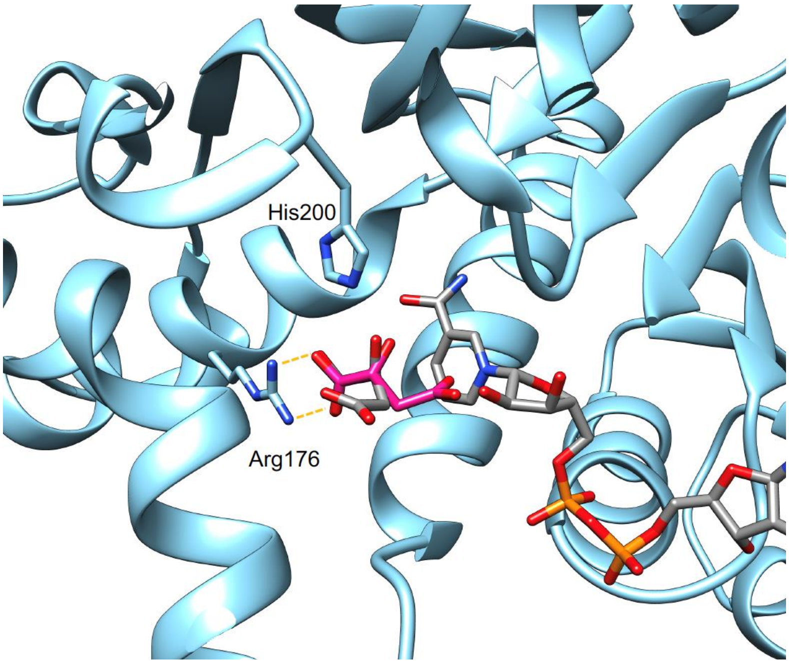

Figure S4. Suggested the mechanism of the substrate specificity. Arg176 and L-malate (gray) formed two hydrogen bonds. Under the assumption that the same hydrogen bonds are retained between D-malate (pink) and Arg176, the structural clash will occur between the other carboxylic group of D-malate and nicotinamide group.

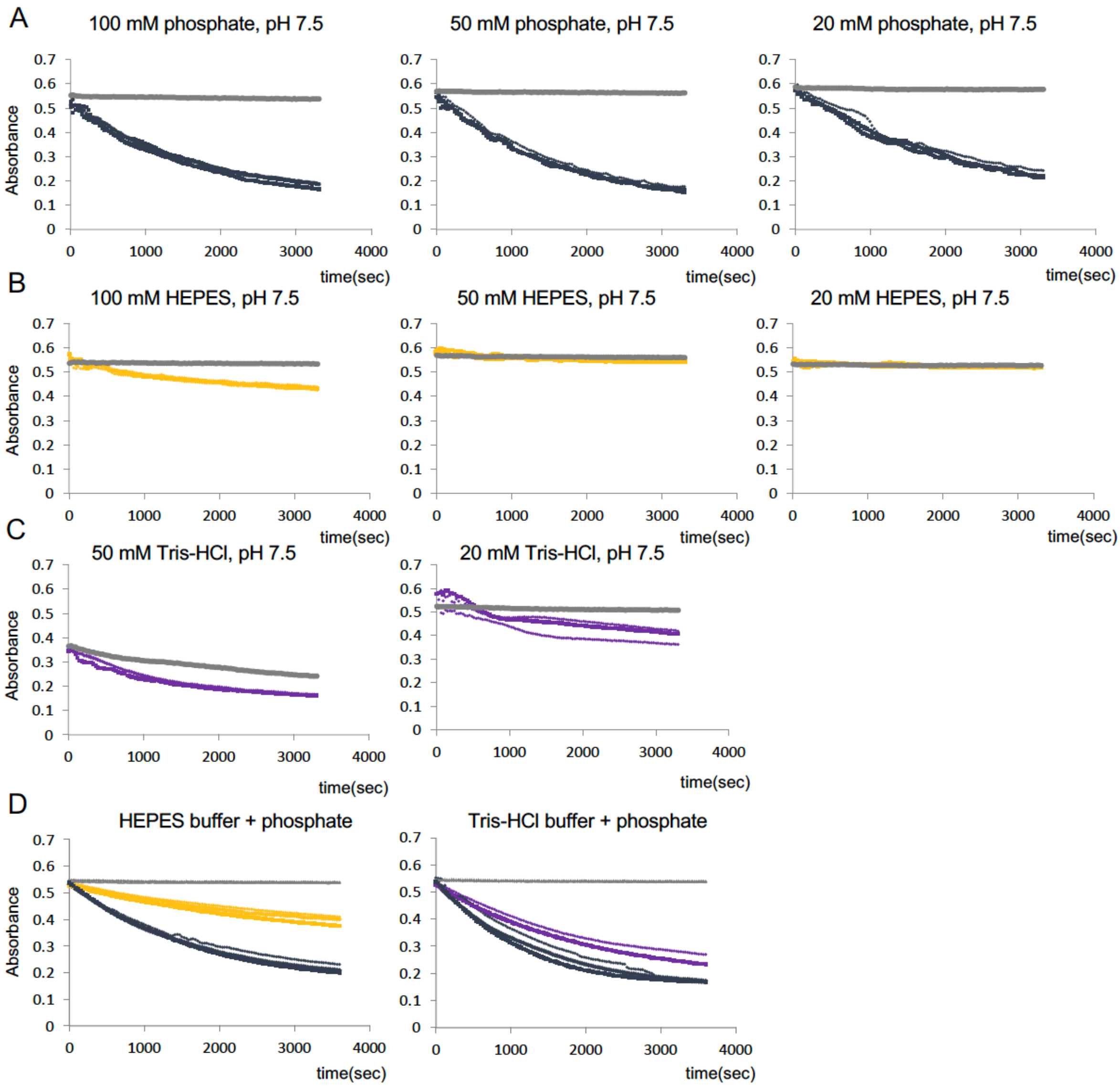

Figure S5. Enzymatic activity of hMDH2 in phosphate, HEPES, and Tric-HCl buffers. In every experiment, the data of reactions without NADH (reference) were shown in gray. The absorbance of NADH at 340 nm were monitored under phosphate (dark blue, A), HEPES (orange, B), and Tris-HCl (purple, C) buffers, respectively. When the final concentration of 20 mM phosphate was added to the reaction solutions with HEPES and Tris-HCl buffers, respectively, the rates of reaction were increased (D).

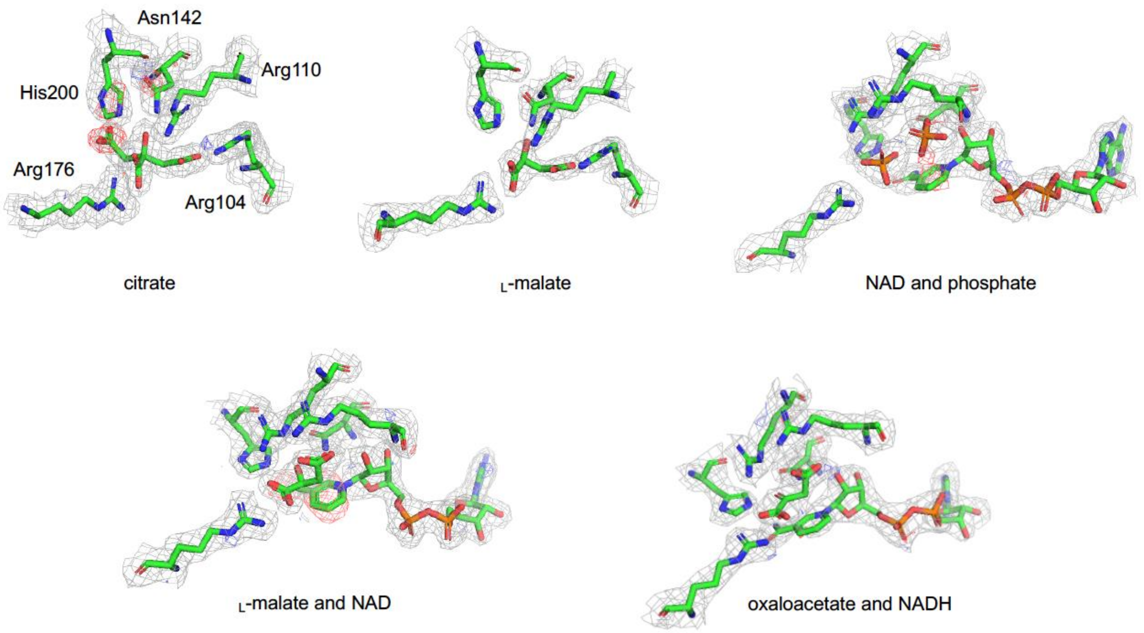

Figure S6. Composite ($2\ F_o - F_c$, gray, contoured at 1σ) and difference ($F_o - F_c$, blue/red, contoured at 3σ) electron density maps of hMDH2. The key residues in substrate binding were indicated.

Table S1. Hydrogen bond interactions across the dimeric interface. Distance was taken from the structure of L-malate and NAD bound hMDH2.

	chain A			chain B		
	Atom	Residue		Atom	Residue	Distance (Å)
1	OD1	67Asp	↔	N	Ser250	2.84
2	OD1	67Asp	↔	OG	Ser250	2.73
3	OD2	67Asp	↔	NZ	Lys241	3.28
4	OD2	67Asp	↔	N	Thr248	2.93
5	OD2	67Asp	↔	OG1	Thr248	3.11
6	OD2	67Asp	↔	N	Leu249	3.02
7	O	His70	↔	OG1	Thr179	2.68
8	OE1	Glu72	↔	ND2	Asn178	2.92
9	OE2	Glu72	↔	OH	Tyr253	2.55
10	OE2	Glu72	↔	NH1	Arg257	2.89
11	NE	Arg176	↔	ND1	His70	3.18
12	ND2	Asn178	↔	OE1	Glu72	2.96
13	OG1	Thr179	↔	O	His70	2.9
14	N	Thr248	↔	OD2	67Asp	2.81
15	OG1	Thr248	↔	OD2	67Asp	3.24
16	N	Leu249	↔	OD2	67Asp	2.99
17	N	Ser250	↔	OD1	67Asp	2.78
18	OG	Ser250	↔	O	67Asp	3.31
19	OG	Ser250	↔	OD1	67Asp	2.6
20	OH	Tyr253	↔	OE2	Glu72	2.63
21	NH1	Arg257	↔	OE2	Glu72	2.92

Table S2. Surface area comparisons of different ligands bound to hMDH2 structures.

Structure	Interfacing molecules	Buried surface area (Å)	Interfacing molecules	Buried surface area (Å)
hMDH2-PO4	A+B	1546.3	A+D	443.1
	C+D	1556.6	B+C	454.4
	(Average)	1551.5	(Average)	448.7
hMDH2-LMR	A+B	1556.2	A+D	456.6
	C+D	1551.4	B+C	456.9
	(Average)	1553.8	(Average)	456.8
hMDH2-CIT	A+B	1535.3	A+D	440.5
	C+D	1531.6	B+C	460.6
	(Average)	1533.4	(Average)	450.6
hMDH2-NAD	A+B	1539.3	A+C	421.1
	C+D	1573.4	B+D	429.2
	(Average)	1556.3	(Average)	425.1
hMDH2-LMR-NAD	A+B	1544.9	A+C	434.1
	C+D	1544.9	B+D	452.3
	(Average)	1549.0	(Average)	443.2
hMDH2-OAA-NAI	A+B	1540.7	A+D	416.8
	C+D	1554.3	B+C	411.8
	(Average)	1547.5	(Average)	414.3

Table S3. Hydrogen bond interactions of hMDH2 and ligands.

Ligand	Atom	Residue	Atom	Hydrogen interaction Distance (Å) hMDH2-LMR chain A	chain B	chain C	chain D	Average	hMDH2-LMR-NAD chain A	chain B	chain C	chain D	Average
L-malate	O4B	Arg 104	NE	2.62	2.74		2.74	2.70	2.79	2.92	2.70	2.83	2.81
	O4A		NH2	2.72		2.82	2.83	2.79	2.77	2.66	2.71	2.69	2.71
	O2	Arg 110	NE	2.72	2.86	2.89	2.80	2.82	2.91	2.71	3.05	3.18	2.96
	O4A		NH2	2.78	2.93	2.85	2.97	2.88	2.74	2.97	2.97	2.80	2.87
	O2	Asn 142	ND2	2.73	2.73	2.55	2.72	2.68	2.87	2.96	2.76	2.85	2.86
	O1B	Arg 176	NH1	2.90	2.96	3.05	2.98	2.97	2.77	2.74	2.77	2.77	2.76
	O1A		NH2	2.75	2.74	2.70	2.80	2.75	2.67	2.75	2.75	2.54	2.68
	O2	His 200	NE2	2.72	2.71	2.75	2.71	2.72	2.61	2.65	2.63	2.69	2.65
	O1B		NE2	3.04	2.96	2.91	2.89	2.95			3.09	2.99	3.04

Ligand	Atom	Residue	Atom	hMDH2-OAA-NADI A	B	C	D	Average
Oxaloacetate	O2	Arg 104	NH2	2.85	2.75	2.69	2.62	2.73
	O3	Arg 110	NE	2.97	2.81	2.94	2.66	2.85
	O2		NH2		2.85	3.29	3.14	3.09
	O3	Asn 142	ND2	2.93	2.79	2.51	2.91	2.79
	O5	Arg 176	NH1	2.64	2.83	2.92	2.63	2.76
	O4		NH2	2.88				2.88
	O3	His 200	NE2	2.78	2.84	2.83	2.95	2.85

Ligand	Atom	Residue	Atom	hMDH2-NAD A	B	C	D	Average	hMDH2-LMR-NAD A	B	C	D	Average
NAD	O2A	Gly 35	N	2.80	2.80	2.70	2.69	2.75	2.74	2.82	2.74	2.73	2.76
	O2N	Ile 36	N	2.89	3.02	2.96	2.96	2.96	3.03	2.99	2.95	2.97	2.99
	O2B	Asp 57	OD1	2.50	2.58	2.74	2.65	2.62	2.62	2.69	2.72	2.57	2.65
	O3B		OD2	2.70	2.76	2.74	2.87	2.77	2.94	2.70	2.69	2.60	2.73
	O4B	Gly 101	N	3.24	3.34		3.27	3.28	3.26		3.30		3.28
	O3D	Val 102	O	3.34	3.22	3.02	2.93	3.13	2.73	2.98	2.80	2.74	2.81
	O3D	Asn 117	ND2			3.11		3.11	3.05	3.08	2.96	3.03	3.03
	N7N	Ile 140	O	3.14	3.11	2.99	3.11	3.09	2.84	3.14	2.97	2.75	2.93
	O2D	Asn 142	ND2	3.10				3.10	3.00	3.18	3.13	3.23	3.14
	O3D		N	3.23				3.23					
	N7N	Val 169	O	3.30	3.20		3.22	3.24	3.17	3.11	3.02	3.12	3.11
	O7N	His 200	NE2	2.83		2.76	2.75	2.78		2.73	2.60		2.67

Ligand	Atom	Residue	Atom	hMDH2-OAA-NAI A	B	C	D	Average
NADH	O2A	Gly 35	N	2.76	2.88	2.82	2.70	2.79
	O2N	Ile 36	N	2.99	3.01	2.88	3.07	2.99
	O2B	Asp 57	OD1	2.48	2.64	2.66	2.32	2.53
	O3B		OD2	2.71	2.43	2.49	2.35	2.50
	O4B	Gly 101	N		3.16			3.16
	O3D	Val 102	O	2.84	3.10	2.91	3.09	2.99
	O3D	Asn 117	ND2			3.03		3.03
	N7N	Ile 140	O	3.22	3.03	3.16	3.25	3.17
	O2D	Asn 142	ND2	2.98	3.04		3.19	3.07
	O3D		N		3.31		3.30	3.31

Ligand	Atom	Residue	Atom	hMDH2-CIT A	B	C	D	Average
Citrate	O1	Arg 104	NH2	2.82	2.78	2.82	2.82	2.81
	O2		NE	2.77	2.78	2.79	2.75	2.77
	O6	Arg 110	NH2	2.93	2.91	3.00	3.00	2.96
	O1		NH2	2.98	3.09	3.09	3.08	3.06
	O7		NE	2.99	3.21	3.19	3.13	3.13
	O7	Asn 142	ND2	2.54	2.65	2.60	2.46	2.56
	O5	Arg 176	NH1	2.87	2.96	2.89	2.86	2.90
	O6		NH2	2.70	2.72	2.64	2.68	2.69
	O7	His 200	NE2	2.75	2.78	2.79	2.79	2.78
	O5		NE2	2.96	3.03	3.09	3.07	3.04

Ligand	Atom	Residue	Atom	hMDH2-PO4 A
PO_4^{2-} (1)	O3	Arg 104	NE	2.78
	O2		N	2.91
	O4	Asn 142	ND2	3.20
	O2	Ser 246	OG	2.59
PO_4^{2-} (4)	O1	Asn 142	N	2.98
PO_4^{2-} (12)	O2	Arg 104	NH2	3.31
	O1	Arg 110	NE	3.20
	O3		NH2	2.79
	O3	Arg 176	NH1	2.64
	O1	His 200	NE2	2.86
